# Association of Extracellular Vesicle Protein Cargo with Race and Clinical Markers of Mortality

**DOI:** 10.1038/s41598-019-53640-1

**Published:** 2019-11-26

**Authors:** Nicole Noren Hooten, Minna H. McFarland, David W. Freeman, Nicolle A. Mode, Ngozi Ezike, Alan B. Zonderman, Michele K. Evans

**Affiliations:** 1 Laboratory of Epidemiology and Population Science, National Institute on Aging, National Institutes of Health, 251 Bayview Boulevard, Baltimore, MD 21224 USA; 2 Present Address: University of North Carolina at Chapel Hill Department of Neuroscience, Chapel Hill, NC USA; 3 Present Address: University of Utah School of Medicine, Salt Lake City, UT USA

**Keywords:** Mechanisms of disease, Diagnostic markers

## Abstract

Differential mortality rates remain a significant health disparity in the United States, suggesting the need to investigate novel potential molecular markers associated with mortality. Extracellular vesicles (EVs), including exosomes, microvesicles and apoptotic bodies, are lipid-bound vesicles secreted by cells into the circulation. EVs mediate intercellular communication by shuttling functional signaling molecules as cargo. EV characteristics by race in the context of mortality risk factors have not been described. We isolated plasma EVs from a cross-sectional cohort of African Americans (AA) and whites and found no significant differences in EV size, distribution or concentration between race or by sex. However, EV cargo showed increased levels of phospho-p53, total p53, cleaved caspase 3, ERK1/2 and phospho-AKT in white individuals compared to AAs. phospho-IGF-1R levels were significantly higher in females compared to males. EV concentration was significantly associated with several clinical mortality risk factors: high-sensitivity C-reactive protein (hsCRP), homeostatic model assessment of insulin resistance (HOMA-IR), alkaline phosphatase, body mass index, waist circumference and pulse pressure. The association of EV proteins with mortality markers were dependent on race. These data suggest that EV cargo can differ by race and sex and is associated with mortality risk factors.

## Introduction

Emerging evidence indicates that extracellular vesicles circulating in blood may be valuable indicators of health and disease^1–3^. These nanosized (30–400 nm) membrane-enclosed vesicles contain important cargo including proteins, lipids and nucleic acids that can be delivered to recipient cells^4,5^. EVs are thought to be intercellular and inter-organ communicators. EVs is a broad term encompassing vesicles in three main categories: exosomes, released through the endosomal/multivesicular body system; microvesicles, shed from the plasma membrane; and, apoptotic bodies, released from dying cells^3,6^. Given the overlap in the size, density and cargo, these vesicles are collectively termed EVs^4,5^.

EVs have been studied in various pathophysiological conditions and diseases, including type 2 diabetes, cardiovascular and neurodegenerative diseases, and cancer^1,7–10^. In addition to their role in pathological situations, EVs are important mediators in maintaining tissue homeostasis^3^. Previously we reported that human aging alters EV concentration, internalization by immune cells and protein cargo^11^. Yet there remain many unexplored questions about EVs in the context of normal human physiology and pathology. Furthermore, there are limited data regarding whether EV characteristics are altered by demographics factors.

Substantial progress has been made in improving health outcomes, yet there are large health disparities for different racial groups. Recent CDC data suggest some recent losses in overall longevity. These data indicate mortality among African Americans (AAs) in the United States remains higher than that among whites^12–14^. This is particularly evident for AA men, who have the highest mortality compared to AA women and whites^12,15^. Alarmingly, there has been an increase in mid-life mortality rates^13^, suggesting the need for the development of biological markers that may be indicators of health for middle-aged individuals at risk.

Many clinical measures are associated with all-cause mortality or predict risk of mortality including C-reactive protein (CRP)^16^, red cell distribution width (RDW)^17^, homeostatic model assessment insulin resistance (HOMA-IR)^18^, growth differential factor 15 (GDF15; also known as MIC-1)^19,20^, total white blood cell (WBC) count^21^, serum albumin^22^, serum alkaline phosphatase (ALP), estimated glomerular filtration rate (eGFR), mean cell volume (MCV), triglycerides, cholesterol, elevated blood pressure and elevated body mass index (BMI)^23^. Although progress has been made in identifying and documenting the use of clinical mortality markers, examination of EVs and their cargo in a middle-aged, racially diverse cohort as novel biomarkers of mortality risk could be useful to enhance identification of those at highest risk.

We examined EV characteristics in the context of race and sex in community-dwelling middle-aged AAs and whites as well as their association with previously identified clinical markers of mortality.

## Results

### Characterization of plasma EVs

To gain a better understanding about whether there are changes in EV characteristics by race and sex, we randomly selected a sub-cohort of participants from the Healthy Aging in Neighborhoods of Diversity across the Life Span (HANDLS) study. These participants were AA and white males and females who were middle-aged between 40–55 years old. Each demographic group contained 25 individuals for a total of 100 individuals (Table 1).

**Table 1:** Demographic and clinical characteristics of the EV cohort.

	AA Male	AA Female	W Male	W Female	*P* value
N	25	25	25	25	
Age	48.26 ± 3.33	48.51 ± 4.9	48.21 ± 4.64	47.17 ± 5.59	0.494
BMI	28.01 ± 6.29	35.54 ± 9.94	31.15 ± 8.15	30.99 ± 6.46	0.016
Cholesterol	199.56 ± 4.68	193.64 ± 35.95	188.96 ± 32.71	196.44 ± 36.64	0.377
Smoking Status (n)	11	4	8	12	0.011

Mean (SD) is shown for variables. Smoking status includes current smokers and was missing for 8 individuals. *P* value for interaction term of race x sex from either an ANOVA (Age, BMI, Cholesterol) or logistic regression (Smoking Status). AA, African American; W, white; BMI, body mass index.

**Figure 1:**
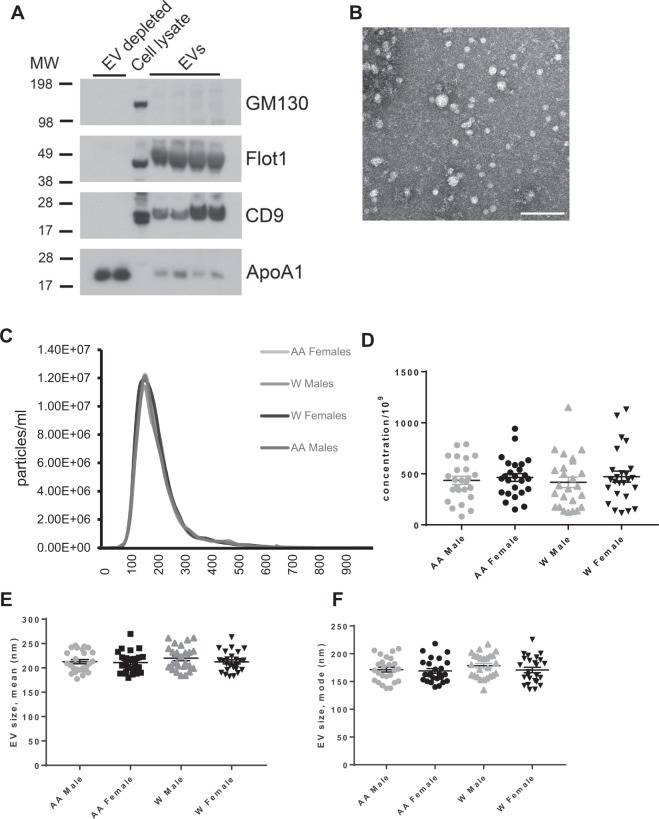
EV characteristics across different demographics. (**A**) Two EV-depleted samples as negative controls, cell lysate, and plasma EV samples were lysed with MPER, analyzed by SDS-PAGE and probed for the known EV markers CD9 and Flotillin1 (Flot1) and the purity markers GM130 and Apolipoprotein A1 (ApoA1). Full immunoblots are in Supp. Fig. 2. (**B**) Electron microscopy images of plasma EVs scale bar = 200 nm. (**C**) EV size distribution, (**D**) concentration, (**E**) size mean, and (**F**) size mode were analyzed across each demographic using Nanoparticle Tracking Analysis (NTA). Two-way ANOVAs tested the association of sex and race with EV size mean, size mode or concentration. Lines indicate the mean and bars indicate the standard error of the mean. AA, African American; W, white.

We isolated EVs from plasma and analyzed isolated EVs according to the minimal information for studies of EVs (MISEV) guidelines from the International Society for Extracellular Vesicles^24^. Plasma EVs were analyzed by immunoblotting against known EV markers CD9 and Flotillin-1 (Fig. 1A). EV markers were enriched in plasma EV samples but absent in EV-depleted samples. Apolipoprotein A1 and GM130 were used as markers for purity. Furthermore, electron microscopy images showed clear rounded intact membrane-bound vesicles in the size range of EVs (Fig. 1B). The size range was further validated using nanoparticle tracking analysis (NTA). NTA showed EVs with a typical size distribution of EVs isolated from plasma with a peak of approximately ~150 nm (Fig. 1C). Previously it has been reported that there are EV size variations between electron microscopy and NTA^25^.

### EV concentration and size with race and sex

Differences in circulating EV concentration have been observed in ovarian and lung cancer, type 2 diabetes mellitus and with human age^8,11,26,27^. However, little is known about whether demographics alter EV characteristics. Therefore, we wanted to test whether there were changes in EV concentration associated with race and sex. Two-way ANOVAs of race and sex did not identify any significant differences for EV concentration, size distribution, mean EV size or EV size mode (Fig. 1C–F).

### Differences in EV protein cargo with race and sex

**Figure 2:**
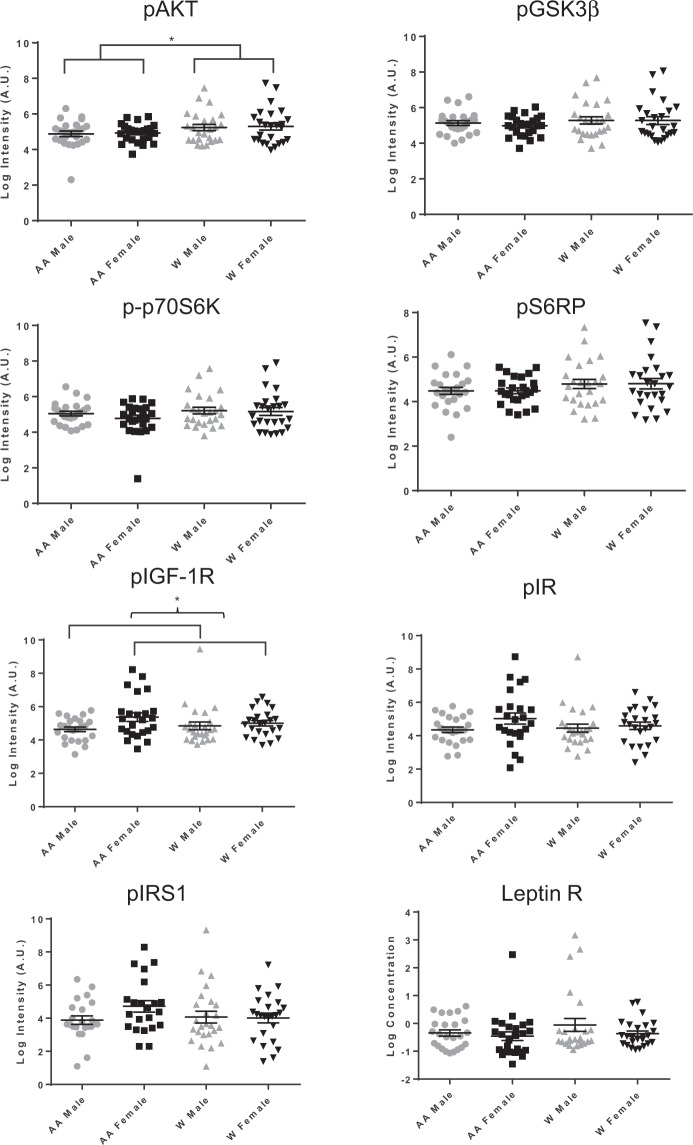
Insulin signaling protein levels in EVs across different demographics. Plasma EVs were lysed and indicated insulin signaling proteins were measured using MesoScale Discovery (MSD) multispot assays or ELISA assays. EV protein levels were log (natural logarithm) transformed and Two-way ANOVAs tested the association of sex and race with each EV protein. *P < 0.05 Lines indicate the mean and bars indicate the standard error of the mean. AA, African American; W, white; p, phospho.

**Figure 3:**
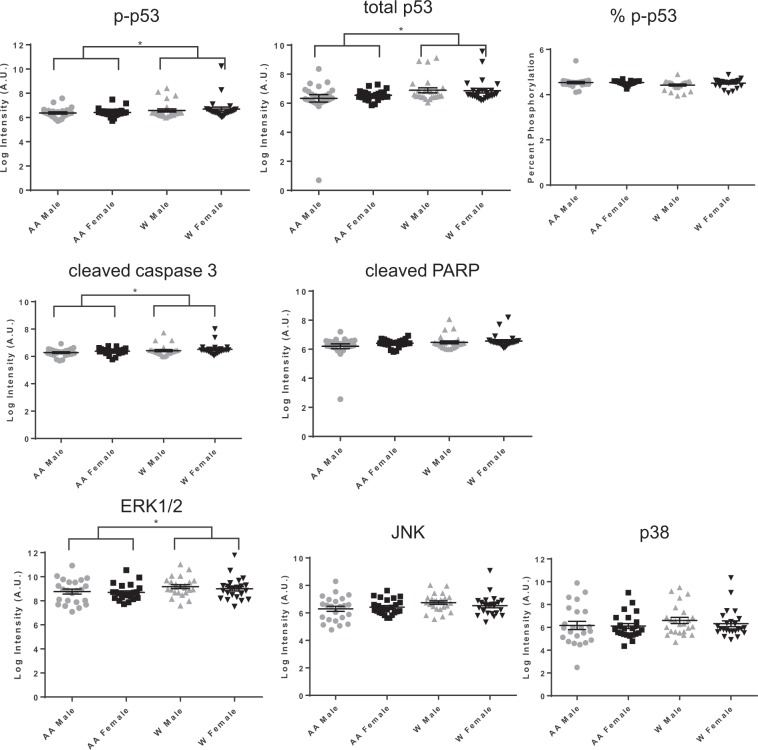
Apoptosis and signaling protein levels in EVs across different demographics. Plasma EVs were lysed and the indicated apoptosis-related proteins and signaling proteins were quantified using MesoScale Discovery (MSD) multispot assays. EV protein levels were log (natural logarithm) transformed and Two-way ANOVAs tested the association of sex and race with each EV protein. Lines indicate the mean and bars indicate the standard error of the mean. % p-p53 was calculated by using the p-p53 value divided by the amount of total p53. *P < 0.05 AA, African American; W, white; p, phospho.

We next analyzed whether EV cargo, including vesicle proteins, were altered in EVs isolated from AA males, AA females, white males and white females. ELISAs were used to measure the phosphorylated forms of proteins involved in the insulin signaling pathway. We focused on this pathway as we previously reported that insulin signaling proteins were present in EVs and were affected by the presence of diabetes mellitus^8^. These proteins included: phospho-p70S6K (Thr389), phospho-S6RP (Ser240/244), phospho-GSK3β (Ser9), phospho-AKT (Ser473), phospho-IR (Tyr), phospho-IGF-1R (Tyr), and phospho-IRS-1 (Tyr) (Fig. 2). In addition, leptin receptor levels were also assessed. We also analyzed apoptosis proteins that we previously found were present in EVs and altered with human aging^11^ including: cleaved PARP (Asp214), total p53, phospho-p53 (Ser15), and cleaved caspase-3 (Asp175) (Fig. 3). We also measured the levels of signaling proteins ERK1/2, p38 and JNK (Fig. 3), as ERK1/2, p38 and JNK play critical roles in mediating a myriad of physiological and pathophysiological processes including proliferation, differentiation and inflammation.

EVs from whites had significantly higher levels of phospho-AKT, phospho-p53, total p53, cleaved caspase 3 and ERK1/2 levels (Figs. 2 and 3). Comparing males to females, there were significantly higher levels of EV-associated phospho-IGF-1R in females compared to males (Fig. 2). The ANOVA results indicated that there was a significant difference in phospho-IGF-1R between males and females overall (*P* = 0.036) but not a significant interaction of sex and race (*P* = 0.167). Thus, the difference between males and females was significant across both races, and not differential between the races. Given the higher BMI for AA females in our cohort (Table 1), we also examined whether BMI accounted for any of the differences in EV protein levels. There was not a significant correlation between BMI and phospho-IGF-1R for either the entire sample (r = −0.009, p = 0.933) or just for AA women (r = −0.143, p = 0.504) indicating that differences in BMI among the sex by race groups did not account for the differences observed in phospho-IGF-1R. In addition, we performed a sensitivity analysis by adding BMI into the ANOVAs examining each protein by sex and race. The significant results were not altered, indicating that BMI does not account for differences observed in the EV protein levels by either race or sex.

### Association of EV concentration and cargo with traditional mortality markers

We wanted to analyze the relationship of EV concentration and protein cargo to known traditional clinical markers that epidemiologic studies have shown are risk factors for mortality. First, we used linear regression to analyze the relationship of EV concentration with race and various clinical measurements. Given the differences between various EV proteins levels between AAs and whites, we included race in our linear regression models. Significant associations with various clinical markers of mortality are listed in Table 2. No significant associations were found between some mortality markers with any EV characteristic including: RDW, GDF15, WBC, serum albumin, and serum insulin. Therefore, these markers are not listed in Table 2. EV concentration was significantly, and positively, associated with hsCRP, homeostatic model assessment of insulin resistance (HOMA-IR), which quantitively measures insulin resistance, serum ALP, BMI, waist circumference and pulse pressure (Table 2A).

**Table 2:** Linear mixed model regression of EV concentration and protein levels on clinical markers of mortality.

A	EV concentration	CRP	HOMA-IR	ALP	BMI	Waist Size	Pulse Pressure	Coefficient
		51.35	42.72	3.168	5.773	3.77	4.61	
		*P* = 0.01	*P* = 0.047	*P* = 0.011	*P* = 0.041	*P* = 0.007	*P = *0.04	P value
**B**	EV protein							
		**Chol**						
	p-p70S6K	−0.007						
		*P* = 0.003						
	pS6RP	−0.007						
		*P* = 0.003						
	pAKT	−0.006						
		*P* = 0.007						
		**Glucose**						
	pIR	−1.878						
		*P* = 0.035						
		**Creatinine**						
	Cleaved PARP	−0.729						
		*P* = 0.007						
	Total p53	−0.879						
		*P* = 0.048						
		**Chol**						
	p38	−0.010						
		*P* = 0.009						
	JNK	−0.004						
		*P* = 0.024						
		**Chol**	**Pulse Pressure**					
	ERK1/2	−0.005	−0.02					
		*P* = 0.028	*P* = 0.05					

Estimated coefficients and the associated P values for separate linear regressions of each EV characteristic on each health measurement accounting for race. Only significant associations are shown. p; phospho; hsCRP, high-sensitivity C-reactive protein; HOMA-IR, homeostatic model assessment insulin resistance; ALP, alkaline phosphatase; BMI, body mass index; Chol, cholesterol.

We also examined the relationship between EV protein levels and these clinical markers of mortality accounting for race. Serum cholesterol and serum glucose were inversely associated with the levels of various insulin signaling proteins in EVs (Table 2B). Cholesterol also had a significant negative relationship with p38, JNK and ERK1/2 levels in EVs. Pulse pressure was also significantly associated with ERK1/2 levels in EVs. Creatinine was negatively associated with apoptosis-related proteins in EVs.

**Figure 4:**
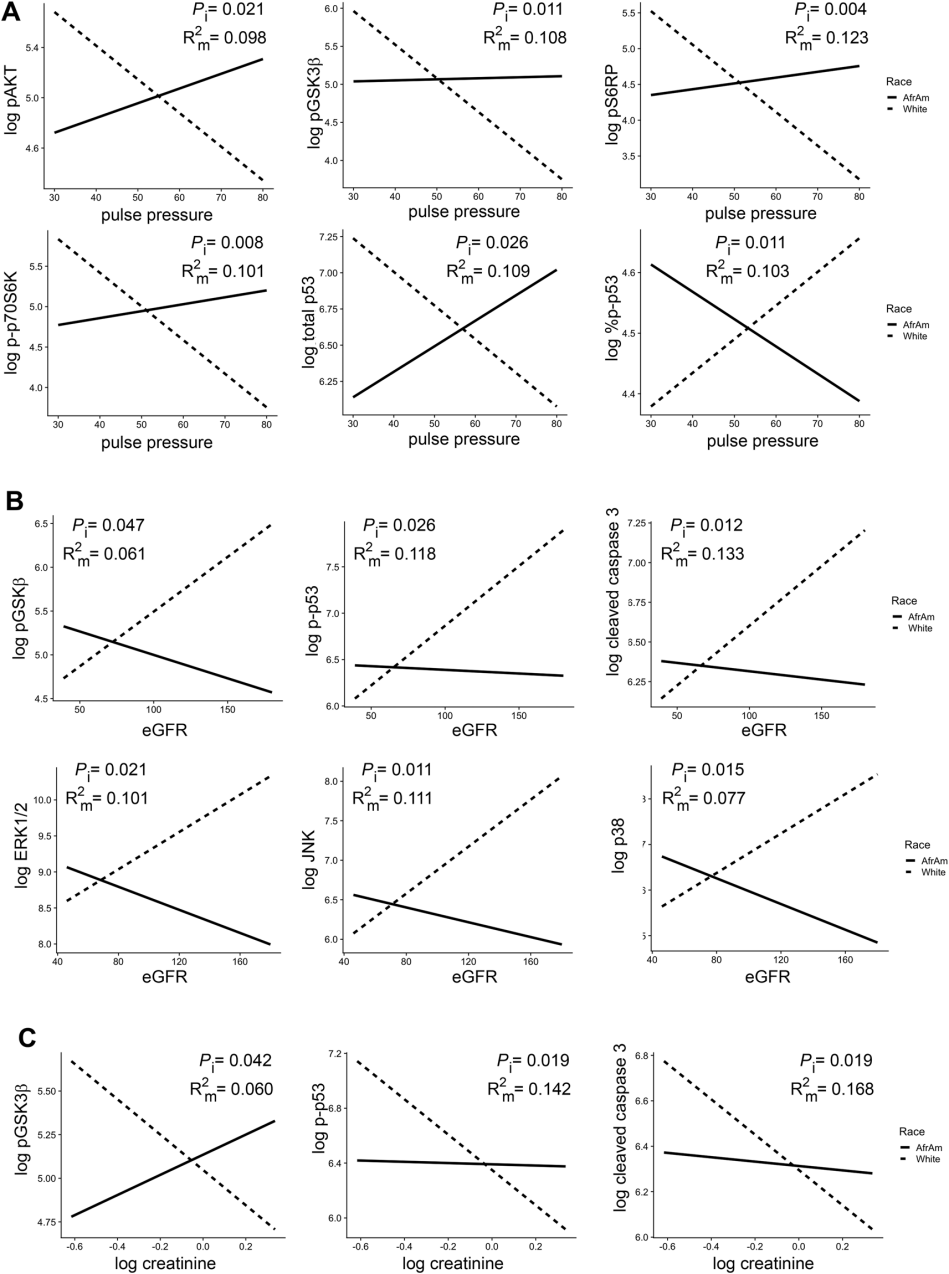
Significant interactions of EV proteins and pulse pressure, eGFR and creatinine and race. Linear regression was used to analyze each EV protein on the interaction of either (**A**) pulse pressure, (**B**) eGFR, and (**C**) creatinine, and race. *P* values for the interactions (*P*_i_) and the R^2^ for the model (R^2^_m)_ are indicated. Solid lines = African Americans (AfrAm) and dashed lines = whites. eGFR; estimated glomerular filtration rate.

Given that the levels of several EV proteins differed by race (Figs. 2, 3), we performed separate linear regressions of each EV variable on the interaction of each mortality marker and race. Several different EV proteins showed a significant race interaction for pulse pressure and eGFR. Interestingly, for pulse pressure, white participants had decreased protein levels with high pulse pressure for all significant proteins except phospho-p53 where the relationship was reversed (Fig. 4A). AAs had increased or stable protein levels with higher pulse pressure except for phospho-p53 where again the relationship was reversed, with high pulse pressure being associated with lower protein levels (Fig. 4A). For eGFR, phospho-GSK3β, phospho-p53 and cleaved caspase 3 EV levels were all increased in whites with high eGFR. eGFR ≥60 mL/min/1.73 m^2^ indicates normal renal function. AAs had decreased protein levels with increasing eGFR levels (Fig. 4B). These data suggest that there are differences in EV levels of these cargo for AAs and whites with normal renal function. Consistent with this idea, creatinine also showed a significant race interaction for phospho-GSK3β, phospho-p53 and cleaved caspase 3 EV levels (Fig. 4C).

**Figure 5:**
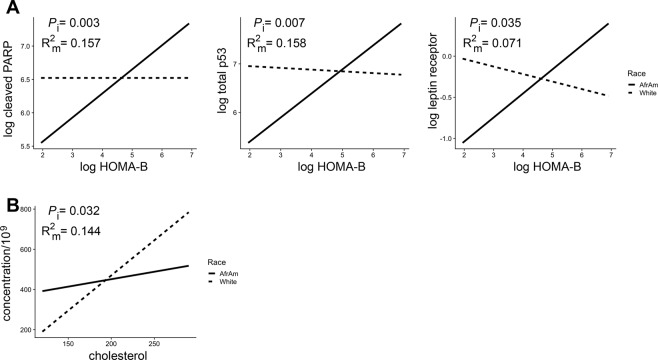
Significant interactions of EV proteins and concentration and HOMA-B, cholesterol and race. Linear regression was used to analyze each EV protein or EV concentration on the interaction of (**A**) HOMA-B and (**B**) cholesterol, and race. *P* values for the interactions (*P*_i_) and the R^2^ for the model (R^2^_m)_ are indicated. Solid lines = African Americans (AfrAm) and dashed lines = whites. HOMA-B, homeostatic model assessment of β-cell function.

An interaction with race was also observed for homeostatic model assessment (HOMA) of β-cell function (HOMA-B), which quantitively measures β cell function, and EV levels of cleaved PARP, total p53 and the leptin receptor (Fig. 5A). These 3 EV proteins all showed a positive association with HOMA-B in AAs and either no association or a slightly negative association in white participants. Cholesterol was the only mortality marker that had a differential association with EV concentration by race (Fig. 5B) with white participants having a stronger positive association between EV concentration and cholesterol level compared to AA participants. There were other EV protein level interactions with race and cholesterol, MCV, ALP, LDH, and MAP (Supp. Fig. 1). These data indicate that the relationship of EV characteristics with many mortality markers differs by race.

## Discussion

Here we analyzed EV concentration and protein levels in a cross-sectional sub-cohort of community-dwelling middle-aged individuals to investigate how demographics may alter EV characteristics as well as the association between EVs and epidemiologic markers of mortality. We found that race and sex had no effect on EV concentration or size. However, race and sex did influence EV cargo. There were significantly higher EV levels of phospho-p53, total p53, cleaved caspase 3, ERK1/2 and phospho-AKT in white individuals compared to AAs. Phospho-IGF-1R was higher in EVs from females compared to males. Furthermore, EV concentration was associated with several markers of mortality including hsCRP, HOMA-IR, ALP, BMI, waist circumference and pulse pressure. EV protein levels were associated with several epidemiologic markers of mortality.

These are important findings given that most studies focus on EVs in the context of disease^1,7–10^. If EVs are to be utilized as a biomarker for the diagnosis and prognosis of disease or treatment outcomes, it is important to further characterize EVs in population-based studies of community-dwelling individuals from diverse racial backgrounds. Thus far, there are limited studies that have examined the association of EVs with demographic factors or routinely obtained clinical measurements. Previously, we analyzed circulating EVs in a cross-sectional and longitudinal cohort of AA and white men and women to examine EV changes during human aging^11^. We found that plasma EV concentration decreases with aging^11^. Using an EV array, another study analyzed the association of various EV proteins with smoking status, age and gender^28^. This cohort consisted of men and women of European descent aged 40–69 who were smokers and non-smokers. There were no sex-based differences in EV protein levels but smoking and age did appear to influence several EV protein levels^28^.

Importantly, we found that several EV-associated proteins were different between AAs and whites. Lower levels of EV-associated phospho-AKT, phospho-p53, total p53, cleaved caspase 3 and ERK1/2 were found in AAs compared to whites. Previously we reported a decrease in levels of apoptosis related-proteins (including p53) with human age^11^. EVs have been reported to convey both anti-apoptotic and apoptotic signals depending on the cellular context and stimuli^3^. One idea is that the presence of these proteins in circulating EVs may reflect the apoptotic status of tissues. Given the lower levels of EV-associated apoptotic proteins with human age, it is interesting to speculate that the lower level of apoptotic proteins in the EVs of AAs may be a potential biomarker for accelerated aging and risk for premature mortality of this group. In support of this idea, we found that many EV-associated apoptotic proteins differed in their relationship to mortality markers by race. This included the association of phospho-p53 with eGFR and creatinine; total p53 with HOMA-B and pulse pressure; percent of phospho-p53 with LDH and pulse pressure; cleaved caspase 3 with MAP, eGFR and creatinine and cleaved PARP with HOMA-B and MAP.

Interestingly, both cleaved caspase 3 and phospho-p53 levels have been associated with kidney disease^29–31^. This could be particularly relevant given that AAs are at highest risk for End Stage Renal Disease. For example, phospho-p53 has been associated with DNA damage and inflammation in rodent models of acute kidney injury and diabetic kidney disease^29–32^. Higher levels of cleaved caspase 3 have also been reported in these rodent models^29–31^. It should be noted that these models of renal injury and kidney disease report on levels of these proteins directly from renal tissue. It would be interesting to examine whether circulating EV levels of caspase 3 and phospho-p53 also increase in these rodent models after kidney injury.

Here we report decreased levels of the activated form of AKT in AAs. Interestingly, in a cohort of individuals who were either euglycemic or with type 2 diabetes we found that with higher levels of HOMA-B there were decreasing levels of phospho-AKT. These data indicate that with worse β-cell function there were lower levels of activated AKT in EVs. As AAs are disproportionally affected by diabetes mellitus and are more likely to suffer from diabetes-associated complications, it is important to identify additional markers that may be useful for identifying at risk populations who bear a disproportionate risk of the disease and its morbidities. In this regard, the association of phospho-AKT and other activated forms of insulin signaling proteins with pulse pressure differed by race.

Little is known about ERK1/2 levels in EVs. However, it is known that upstream regulators of ERK1/2, including mutant KRAS, can be functionally transferred within EVs and dramatically effect EV proteomic composition^33,34^. ERK1/2 is an important modulator of growth factor-mediated cell proliferation and this pathway is often disrupted in conditions such as neurological disease and cancer^35^. In our study, ERK1/2 levels were associated with cholesterol and pulse pressure. Furthermore, the relationship between ERK1/2 and eGFR differed by race. It will be important in the future studies to address whether changing levels of EV-associated ERK1/2 may be a potential indicator of health status.

Little is known about the relationship of EVs to clinical markers of mortality. The strength of our study lies in examining these relationships. We found a strong positive association of EV concentration to hsCRP, HOMA-IR, ALP, BMI, waist size and pulse pressure. EV proteins related to signaling and insulin signaling were associated with cholesterol, glucose or pulse pressure. Creatinine was associated with multiple EV-associated apoptotic proteins. Another strength of our study is our cohort, which consists of middle-aged individuals. Recent epidemiologic data reveals a marked increase in mid-life mortality rates^13^, yet most mortality biomarker studies are conducted using elderly, white populations. To address this, we designed our cohort to contain middle-aged AAs and whites to examine both sex and race differences and to decipher whether EVs and their cargo have associations with clinical markers of mortality. Interestingly, EV concentration was positively associated with mortality markers and several EV-proteins were negatively associated with mortality markers. One limitation of our study includes our cohort size, which is consistent with other EV studies using human biofluids^8,11,36–39^, but is less than many epidemiological studies that examine markers of mortality. This does hinder our capacity to examine multiple covariates in our regression models in order to maximize our power to detect differences.

Nevertheless, this study sheds new light on the clinical relevance of EVs in middle-aged community-dwelling individuals. As most studies have focused on EVs in the context of disease, our data provide important information that will hopefully aid in designing and interpreting data for the development of EV-based diagnostics.

## Materials and Methods

### Cohort design

Participants were from the Healthy Aging in Neighborhoods of Diversity across the Life Span (HANDLS) study of the National Institute on Aging, Intramural Research Program at the National Institutes of Health (NIH)^40^. The Institutional Review Board of the National Institute of Environmental Health Sciences, NIH, approved this study and all participants provided written informed consent. All experiments were performed in accordance with relevant guidelines and regulations. We selected 100 participants in a factorial cross of sex and race; AA males (n = 25), AA females (n = 25), white males (n = 25), and white females (n = 25). More details on demographics and clinical characteristics of the cohort can be found in Table 1. Selected participants were 40–55 years old at their blood draw and free from the following conditions: lupus, rheumatoid arthritis, HIV/AIDS, Hepatitis B/C, psoriasis, multiple sclerosis (MS), cancer, Alzheimer’s disease, and diabetes mellitus.

Participants underwent a medical examination and a structured medical history interview. Body mass index (BMI = weight[kg]/height[m^2^]) was calculated from measured height and weight. Smoking use was defined as current or non-user. Blood samples were obtained, and serum was assayed by Quest Diagnostics (Nichols Institute, Chantilly, VA). Fasting glucose, insulin, cholesterol, creatinine, lactate dehydrogenase (LDH), white blood cell count (WBC), albumin, mean corpuscular volume (MCV), alkaline phosphatase (ALP), and high-sensitivity CRP (hsCRP) were assayed by Quest Diagnostics. Quest Diagnostics measured red cell distribution width (RDW) by automated Coulter DXH 800 hematology analyzer as part of peripheral complete blood count (Beckman Coulter, Brea, CA). GDF15 was measured using an ELISA kit from R & D systems (DGD150).

Homeostatic model assessment (HOMA) of β-cell function (HOMA-B) and HOMA of insulin resistance (HOMA-IR) were calculated based on fasting insulin and glucose levels as previously reported^41^. Blood pressure was taken while seated after a 5-minute rest and averaged for measurements in both arms. Pulse pressure was defined as the numeric difference between systolic and diastolic blood pressure. Mean arterial pressure (MAP) was calculated as one-third the pulse pressure plus the diastolic blood pressure and estimated glomerular filtration rate (eGFR) was calculated using the Modification of Diet in Renal Disease (MDRD) Study equation by race and sex.

### Plasma EV isolation

Plasma was isolated from HANDLS participants as previously described^11^. Plasma (0.4 mL) was thawed on ice and EVs were isolated using ExoQuick™ Exosome Precipitation Solution (System Bioscience Inc.) with some modifications from the manufacturer’s protocol. This isolation procedure provided more reproducible data than differential ultracentrifugation or size exclusion columns^11^ and also allowed for the processing of a large number of human samples.

Plasma was first treated with 0.2 mL Thromboplastin D (Cat#:100354; Fisher Scientific, Inc.), incubated at room temperature for 30 minutes and then 0.3 mL of Dulbecco’s phosphate buffered saline (DBPS) supplemented with protease and phosphatase inhibitors was added. Samples were centrifugation at 3000 × *g* for 20 minutes at 4 °C. The supernatants were collected and mixed with ExoQuick™ (252 μl), incubated for one hour at 4 °C and then centrifuged at 1500 × *g* at 4 °C for 20 minutes. The supernatant was removed and saved for analysis as the EV-depleted plasma fraction. The EV pellet was resuspended in 0.5 mL of nanopure water supplemented with protease and phosphatase inhibitors. An aliquot of the EVs was diluted in DPBS at 1:300 dilution for NTA. A 0.1 mL aliquot of EVs were lysed in MPER at a 1:3 dilution for ELISAs and immunoblotting. All samples were stored at −80 °C until further analysis.

### Immunoblotting

Equal amounts (5 μg) of EVs, CEM (T lymphoblast) cell lysate, and two ExoQuick EV-depleted supernatant samples were analyzed using SDS-PAGE followed by immunoblotting with antibodies against known EV protein markers Flotillin1 (clone EPR6041; Abcam), CD9 (EXOAB-CD9A-1; System Biosciences), and the EV purity markers GM130 (ab52649; Abcam) and Apolipoprotein A1 (ab64308; Abcam). Full immunoblots are in Supp. Fig. 2.

### Electron microscopy

Plasma EVs were isolated as described above and electron microscopy was performed as previously described by the Johns Hopkins University Neurology Microscopy Core^11^. Grids were visualized on a Libra 120 transmission electron microscope at 120 Kv (Zeiss) and images were obtained using a Veleta camera (Olympus).

### Nanoparticle Tracking Analysis (NTA)

EV size and concentration were determined by NTA using a NanoSight NS500 (Malvern Instruments Ltd.) as instructed by the manufacturer’s protocol. For each sample, five 20 second videos were recorded at Camera Level = 14. Analysis was performed using NanoSight Software NTA 3.2 Build 3.2.16 at Detection Limit = 3. Parameters were based on manufacturer’s recommendations including a detection threshold that enabled enumeration of as many particles as possible while ensuring the least amount of background and false positives (<5 blue cross count). All samples were analyzed using the same settings by a single user to enhance concentration measurement accuracy. EV isolation, size and concentration analyses were performed blind. Plasma EV concentration was calculated as described previously^11^.

### ELISA

Plasma EVs were lysed as described above and equal volumes were used to quantitatively measure proteins in the following Meso Scale Discovery kits: pp70S6K (Thr389), pS6RP (Ser240/244), pGSK3β (Ser9), and pAKT (Ser473) by the Akt Signaling Panel II Whole Cell Lysate Kit (Cat#:K15177D-1); cleaved PARP (Asp214), total p53, phospho-p53 (Ser15), and cleaved caspase-3 (Asp175) by the Apoptosis Panel Whole Cell Lysate Kit (Cat#:K15102D-1); pIR (Tyr), pIGF-1R (Tyr), and pIRS-1 (Tyr) by the Insulin Signaling Panel Phospho Protein Kit (Cat# K15151C-2) and ERK1/2, p38 and JNK by the MAP kinase (Total Protein) Whole Cell Lysate Kit (Cat#: K15157D-2). Meso Scale kits were analyzed on a Meso Quickplex SQ 120. Leptin receptor was measured using an ELISA kit (R&D Systems; Cat# DY389).

### Statistics

Protein levels were skewed and thus log (natural logarithm) transformed. ANOVAs were used to examine the relationship among sex and race for EV concentration, size and protein levels. Linear regression models were used to examine the relationship between EV variables with various clinical measurements. hsCRP, serum insulin, serum glucose, GDF15, RDW, HOMA-B, and HOMA-IR values were all log (natural logarithm) transformed for analysis. Clinical variables with significant interactions with race were excluded from Table 2.

## Supplementary information

Supplementary Information

## Data Availability

The datasets generated and analyzed during the current study are available from the corresponding author on reasonable request through the HANDLS website https://handls.nih.gov/.
