# Supplementary material for: Association of Extracellular Vesicle Protein Cargo with Race and Clinical Markers of Mortality
Source: Sci Rep. 2019 Nov 26;9:17582. doi: 10.1038/s41598-019-53640-1 (PMC6879565; doi:10.1038/s41598-019-53640-1)

Supplementary Information: Figures S1,S2

Association of Extracellular Vesicle Protein Cargo with Race and Clinical Markers of Mortality

Nicole Noren Hooten, Minna H. McFarland, David W. Freeman, Nicolle A. Mode, Ngozi Ezike, Alan B. Zonderman, and Michele K. Evans

Supplementary Figure Legends

Supplementary Figure 1. Significant interactions of EV proteins and various markers of mortality and race. Linear regression was used to analyze each EV protein on the interaction of (A) LDH, (B) MAP, (C) ALP, (D) cholesterol, (E) MCV, and race. *P* values for the interactions (P_i) and the R^2 for the model ($R^2_{m)}$ are indicated. Solid lines=African Americans (AfrAm) and dashed lines=whites.

Supplementary Figure 2. Full immunoblots for EV markers. Two EV-depleted samples as negative controls, cell lysate, and plasma EV samples were lysed with MPER, analyzed by SDS-PAGE and probed for the known EV markers CD9 and Flotillin1 (Flot1) and the purity markers GM130 and Apolipoprotein A1 (ApoA1). MW, molecular weight

Suppl. Figure 1

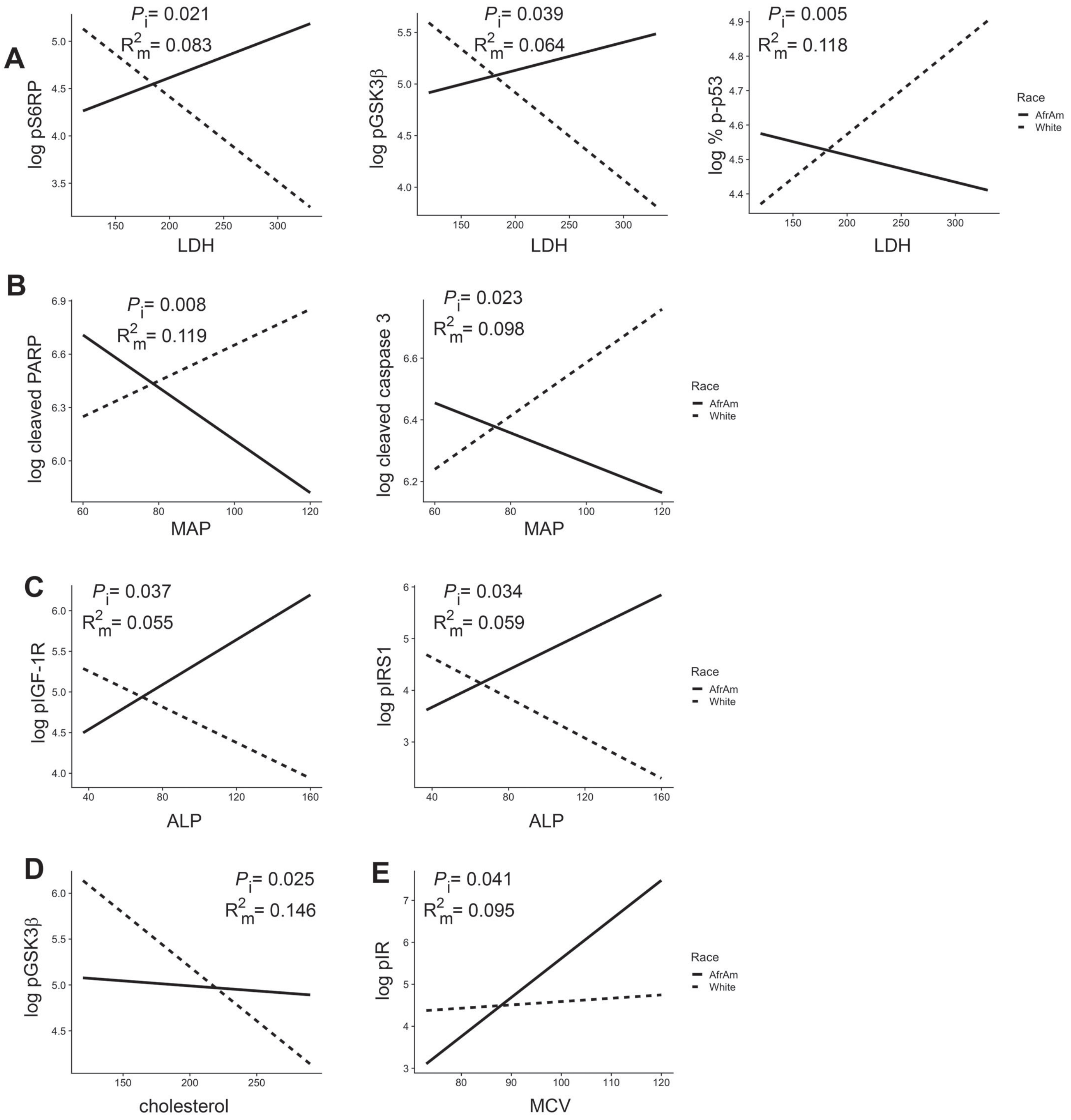

Supp. Figure 2

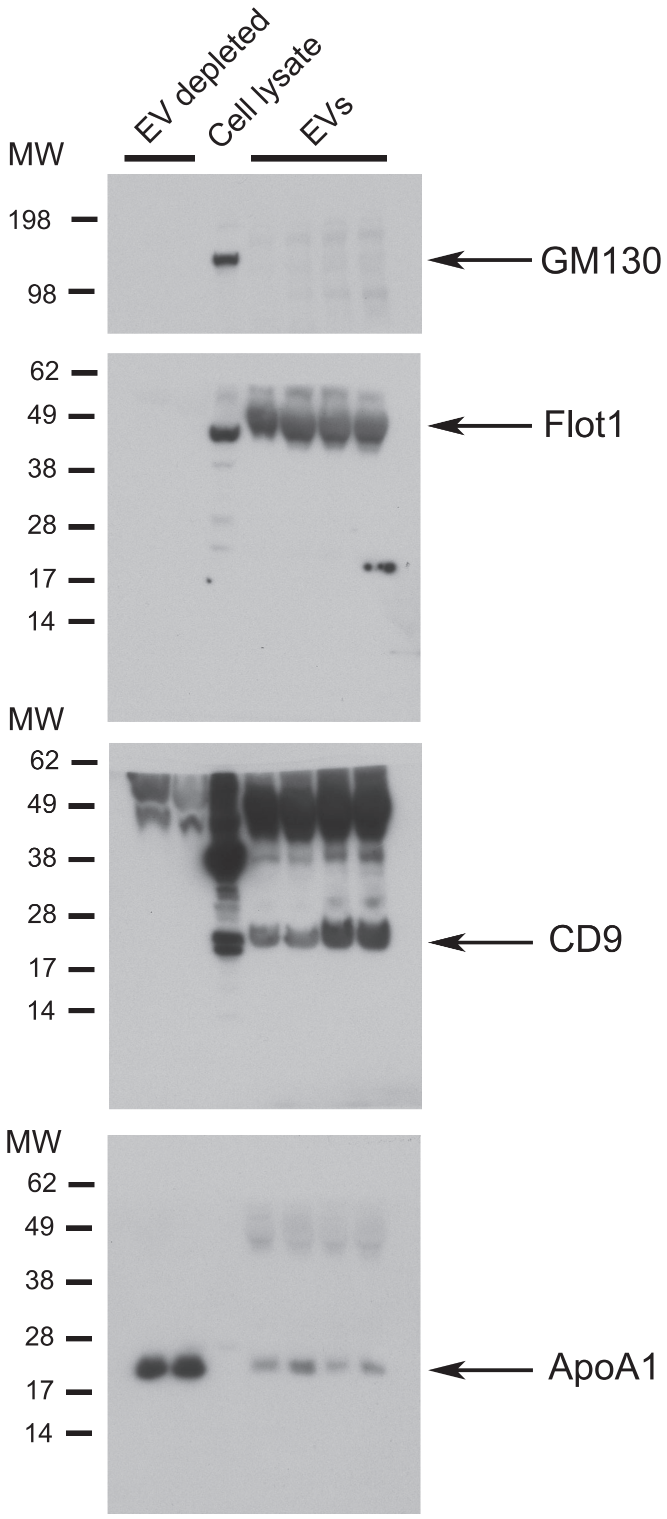

Supplement: Supplementary file 1 — Supplementary Information [file 41598_2019_53640_MOESM1_ESM.pdf]
